# Vancomycin and Oxacillin Co-Resistance of Commensal Staphylococci

**DOI:** 10.5812/jjm.9310

**Published:** 2014-04-01

**Authors:** Ariyo Oludotun Soyege, Adedayo Olajide Ajayi, Elvis Ngwenya, Albert Kotze Basson, Anthony Ifeanyin Okoh

**Affiliations:** 1 Applied and Environmental Microbiology Research Group, Department of Biochemistry and Microbiology, University of Fort Hare, Alice, South Africa; 2 Department of Biochemistry and Microbiology, University of Zululand, Kwadlangezwa, South Africa.

**Keywords:** *Staphylococcus*, Antibiotic resistance, Vancomycin, Oxalocillin

## Abstract

**Background:**

Many disease conditions including Staphylococcal infections are becoming increasingly difficult to treat in South Africa due to the surge of vancomycin-oxacillin resistant strains. How widespread this phenomenon is in commensal isolates in the Nkonkobe municipality in the Eastern Cape Province of South Africa is not known, and considering the high level of immunocompromised individuals in the province, this study couldn’t have come at a better time.

**Objectives:**

The objective of this study is to evaluate the prevalence of vancomycin and oxacillin co-resistance in methicillin-resistant commensal staphylococci in Nkonkobe municipality, South Africa as part of our larger study on the surveillance of reservoirs of antibiotic resistance in South Africa.

**Materials and Methods:**

*Staphylococcus* species were isolated from domestic animals of Nkonkobe municipality, in the Eastern Cape Province of South Africa. The isolates were evaluated for antibiotic susceptibility against a panel of several relevant antibiotics. Specific primer sets were also used for the polymerase chain reaction assay to detect the presence of *mecA* gene as well as *vanA* and *vanB* genes in the genome of resistant *Staphylococcus* species.

**Results:**

A total of 120 *Staphylococcus* isolates were screened, out of which, 32 (26%) were susceptible to both methicillin and vancomycin, while 12 (10%) had co-resistance to the antibiotics, which is still on the high side, both clinically and epidemiologically. Gentamicin (an aminoglycoside) had a relatively high potency against the isolates with 107 (89.17%) of the bacteria being susceptible to it, while 10 (8.33%) were resistant. On the other hand, erythromycin (a macrolide) was active against 72 (60%) of the isolates, while 5 (4.17%) and 74 (61.67%) of them yielded intermediate and resistant responses, respectively. Similarly, 51 (42.5%) of the isolates were susceptible to rifampicin, while 1 (0.83%) and 17 (14.17%) were intermediate and resistant, respectively.

**Conclusions:**

Ten percent of the isolates were positive for *mecA* gene among the vancomycin-oxacillin resistant strains, while *van* gene was not detected in any of the isolates. The data obtained would be useful in clinical control of resistant staphylococcal strains.

## 1. Background

Resistance to antibiotics has become important in clinical control of many diseases and deserves scientific intervention to bring about some control measures. Therefore, the issue of vancomycin-methicillin co-resistance in commensal *Staphylococcus* species in any locality can be summarily tackled by determining the pathogenic nature of aetiologic agents involved and seeking effective antimicrobial agents against such resistant strains. Most staphylococcal infections are caused by *Staphylococcus aureus *(1, 2). This bacterium is the cause of a wide range of pathogenic infections, though also a commensal of human skin and nares. *S. aureus* most commonly causes skin infections such as folliculitis, boils, impetigo and cellulitis, which are limited to a small area on the individual’s skin (2, 3). 

According to Mahajan (4), role of *S. aureus* in pathology in any organ of the body, including the eyes, is not disputed. Furthermore, the acceptance of *S. epidermidis* as a pathogen in different sites of the human body is gradually growing (5). *S. epidermidis* has been reported as the second-most common pathogen in bacteriologically proven cases of urinary tract infection, and novobiocin-resistant micrococci have been claimed to have a special predilection for the urinary tract of human beings (6, 7). Such cocci have also been recovered from lesions of the eye, which regressed when appropriate antimicrobial agents were administered (1, 8).

Generally, various health care organizations worldwide have developed numerous channels in educating the public and assigning research funds to tackle the problems emanated from multi-drug resistant *S. aureus* (9, 10).

## 2. Objectives

In this paper, we reported vancomycin and oxacillin co-resistance in methicillin-resistant commensal staphylococci in Nkonkobe municipality, South Africa as part of our larger study on the surveillance of reservoirs of antibiotic resistance in South Africa.

## 3. Materials and Methods

### 3.1. Isolates Source

The *Staphylococcus* species were obtained from the culture collections of the Applied and Environmental Microbiology Research Group (AEMREG), University of Fort Hare, Alice, South Africa and isolated from ear and nasal swabs of some domestic animals including pigs, cattle, cows and goats in different areas of Nkonkobe municipality in the Eastern Cape Province, South Africa. Thirty isolates from each of pigs, cattle, cows and goats, making a total of 120 *Staphylococcus* isolates, were used for the study. Ear: nasal ratio of 1:1from each of these animal sources was used throughout the study.

### 3.2. Standardization of Inoculums and Inoculation of Plates

The stock cultures were reactivated by subculturing into tubes containing nutrient broth and incubated for 24 hours at 37˚C with shaking and thereafter subcultured onto nutrient agar plates and again incubated for 24 hours at 37˚C. Three to five colonies from each plate were then suspended in tubes containing 5 mL of sterile distilled water and vortexed thoroughly to achieve a uniform suspension. Turbidity of the suspension was compared with that of 0.5 McFarland standards and adjusted as required. The standardized inoculums were used in antibiotic susceptibility tests within 15 minutes. Freshly-prepared Mueller-Hinton agar plates were inoculated with the bacterial suspension using a sterile swab, by which even lawns of the bacteria were produced. Antibiotic discs were then placed on the surface of the agar using sterile forceps and the plates were incubated at 35˚C for 18-24 hours. The zones of inhibition were then measured using a ruler and interpreted using available interpretive charts as shown in Figure 5 (11, 12).

### 3.3. DNA Extraction

Extraction of the bacterial DNA was performed using the boiling method. A fresh colony of the *Staphylococcus* culture was suspended in 500 µL of diethylpyrocarbonate (DEPC)-treated water (DNase-RNase free) and boiled for 10 minutes using a heating block. Thereafter, the suspension was centrifuged at 10000 rpm for 5 minutes. The supernatant, containing the bacterial DNA, was used as a template for subsequent PCR reactions.

### 3.4. Polymerase Chain Reaction Conditions

The initial denaturation step was performed at 94˚C for 5 minutes, followed by 10 cycles of amplification, denaturation at 94˚C for 30 seconds, and extension at 72˚C for 45 seconds. Another set of 25 cycles of amplification, including denaturation at 94˚C for 30 seconds, annealing at 50˚C for 45 seconds and extension at 72˚C for 2 minutes, ending with final extension step at 72˚C for 10 minutes was performed.

### 3.5. Assessment of Putative mecA Gene and van Genes

Previously reported primer sets(13, 14, 15) were used in the polymerase chain reaction (PCR) to detect the presence of *mecA *gene as well as *vanA* and *vanB* genes in the resistant *Staphylococcus* species, shown in Table 1.

**Table 1.** Primers Used for the PCR Detection of *mecA* and *van* Genes in the *Staphylococcus* species

Target Genes	Primer Sequence	Amplicon Size, bp	Annealing Temperature	References
***mecA***		310	50˚C for 45 sec	Geha DJ et al. (13)
	Forward: 5ʹ-GGTCCCATTAACTCTGAAG-3ʹ			
	Reverse: 5ʹ-CCAATTCCACATTGTTTCGGTCTAA-3ʹ			
***VanA***		Negative	51.6˚C for 1 min, 72˚C for 1 min	Dutka-Malen S et al. (15)
	Forward: 5ʹ-GGGAAAACGACAATTGC-3ʹ			
	Reverse: 5ʹ-GTACAATGCGGCCGTTA-3ʹ			
***VanB***		Negative	51.6˚C for 1 min, 72˚C for 1 min	Ramos-Trujillo E et al. (14)
	Forward:5ʹ-GTGCTGCGAGATACCACAGA-3ʹ			
	Reverse: 5ʹ-CGAACACCATGCAACATTTC-3ʹ			

## 4. Results

This study showed the susceptibility patterns of vancomycin-methicillin co-resistance in commensal *Staphylococcus* species in Nkonkobe municipality, Eastern Cape, South Africa. It also helps in the determination of domestic animals as reservoirs after antibiotic resistant strains and their public health implications. As evident from Table 2, about 32 (26.67%) tested isolates were susceptible to both methicillin and vancomycin, while 12 (10%) of the isolates from these sources had co-resistance to the antibiotics, which is still on the high side, both clinically and epidemiologically. Other classes of antibiotics were used for further clarification of this resistance pattern.

Table 3 shows β-lactam antibiotic profile of the *Staphylococcus* isolates. Of the bacterial isolates, 115 were susceptible to meropenem (MEM), while about 90.83% were susceptible to sulbactam-ampicillin (SAM). On the other hand, 93 (77.5%) of the isolates were moderately susceptible to ceftriaxone (CRO), while only 7 (5.83%) were fully susceptible to it and 12 (10%) were resistant. In addition, 10 (8.33%) of the isolates were susceptible to ceftazidime (CAZ), while 22 (18.33%) were susceptible to aztreonam (ATM) and 94 (78.33%) were resistant.

**Table 2.** Profile of Vancomycin-Methicillin Co-Resistance in Commensal *Staphylococcus* Species Isolated From the Nkonkobe Municipality Environment

Methicillin	Vancomycin				
	Susceptible	Moderately Susceptible	Intermediate	Resistant	Resistant Strains, %^a^
**Susceptible**	32	2	6	7	5.83
**Intermediate**	12	-	1	6	5
**Resistant**	10	14	1	12	10

^a^Resistant strains (%),percentage of the isolates that were positive for the resistant genes out of the 120 isolates.

**Table 3.** Susceptibility Profile of *Staphylococcus* Species Isolated From the Nkonkobe Municipality Environment to Some β-lactam Antibiotics ^a^, ^b^

β-lactam Antibiotics	Susceptible Strains	Moderately Susceptible Strains	Intermediate Response	Resistant Strains	Moderately Resistant Strains	Resistant Strains, % ^c^
**CRO, 30 µg**	7 (5.58)	93 (77.5)	-	12 (15.83)	7 (5.58)	15.83
**AUG, 30 µg**	75 (62.5)	-	-	4 (3.33)	-	3.33
**CAZ, 30 µg**	10 (8.33)	19 (15.83)	36 (30)	37 (30.83)	-	30.83
**CTX, 30 µg**	37 (30.83)	52 (43.33)	6 (5)	19 (15.83)	-	15.83
**SAM, 10 µg**	109 (90.83)	-	2 (1.67)	13 (10.83)	-	10.83
**PEN-G, 10 Units**	53 (44.17)	-	-	53 (44.17)	-	44.17
**MEM, 10 µg**	115 (95.83)	-	1 (15.83)	4 (3.33)	-	3.33
**ATM, 30 µg**	22 (18.33)	1 (0.83)	-	94 (78.33)	-	78.33

^a^ Abbreviations: ATM- Aztreonam; AUG, augmentin; CAZ, ceftazidime; CRO, ceftriaxone CTX, cefotaxime; MEM, meropenem; PEN-G, penicillin G; SAM, sulbactam ampicillin.

^b^ Data are presented in No. (%).

^c^ Resistant strains (%), percentage of isolates that were positive for the resistant genes out of the 120 isolates.

In addition to the assessment, 107 (89.17%) of the isolates were susceptible to the aminoglycoside (gentamicin), while 10 (8.33%) were resistant. In comparison, 72 (60%) of the isolates were susceptible to the macrolide (erythromycin) while 5 (4.17%) and 74 (61.67%) showed intermediate and resistant responses, respectively. Similarly, 51 (42.5%) of the isolates were susceptible to the anti-tuberculosis agent (rifampicin), while 1 (0.83%) and 17 (14.17%) showed intermediate and resistant responses, respectively. Molecular detection of the *mecA *gene revealed the expected amplicon size of 310 base pairs (bp) in the positive isolates. Molecular detection of *mecA *and *van* genes revealed that 10% of the isolates were positive for *mecA *gene, among the isolates with co-resistance to both vancomycin and oxacillin (Table 2). *Van* gene was not detected in any of the isolates.

## 5. Discussion

This study showed the range of vancomycin-methicillin co-resistance in commensal *Staphylococcus* species among other classes of antibiotics tested in Nkonkobe municipality and its public health implications. Recent times saw a burgeoning literature on some characteristics that used to be the exclusive preserves of clinical staphylococcal isolates, but are now in the commensal subgroups. Typical examples include the formation of thick, multilayered biofilms on inert surfaces, such as polymers or metals, known to be attributes of nosocomial pathogens (16) and pronounced resistance against many of today’s commonly-used antibiotics including methicillin.

According to Bignardi et al. (17) and Wielders et al. (18), most clinical isolates of methicillin-resistant *S. aureus* harbor *mecA *gene, which encodes the production of PBP2a, a modified penicillin-binding protein with low affinity for β-lactam antibiotics (19). However, the emergence of resistance *in vitro*, as a result of mutations, during subculture on media with increasing methicillin concentrations, has also been documented (20, 21). The present study in Nkonkobe municipality showed the prevalence of resistant strains of *Staphylococcus* species, which also corroborates with previous investigations by Moodley et al. (22) who gave an account of clinical methicillin-resistant *S. aureus* (MRSA) isolates from various infection sites collected throughout South Africa. Similarly, Science News by United Press International (UPI) (23), also demonstrated the effort of a US pharmaceutical company to find treatment solutions for the worst staphylococcal infections in the soil of South Africa.

The study of Domaracki et al. (24) showed that vancomycin was the drug of choice for most methicillin-resistant *Staphylococcus* infections, and therefore, the recent emergence of decreased vancomycin susceptibility in methicillin-resistant staphylococci presents a significant clinical problem. Furthermore, reduced susceptibility to vancomycin in *Staphylococcus* species appears to occur on exposure to vancomycin and under selective pressure, rather than by gene transfer as in enterococci (24, 25). *In vitro* experiments have demonstrated that selective pressure can produce vancomycin resistance, but have also revealed that increases in vancomycin resistance can induce concurrent decreases in resistance to β-lactams in both methicillin-resistant coagulase-negative staphylococci (MRCNS) and methicillin-resistant *S. aureus *(MRSA). 

The study of Domaracki et al. (24) further showed that clinical isolates of vancomycin-susceptible MRCNS and MRSA became increasingly susceptible to oxacillin when grown in the presence of a sub-MIC of vancomycin. However, the present study specifically demonstrated the potency of the β-lactam antibiotics, meropenem (MEM), sulbactam-ampicillin (SAM) and gentamicin (an aminoglycoside) to be very effective for the control of multiple resistant commensal staphylococci in Nkonkobe municipality, South Africa. Findings of this study could be a good guide in infectious diseases control, especially with respect to *Staphylococcus* infections in the community.
